# Incidentalomas Among Healthy Nephrology Fellow Volunteers at POCUS Workshops: A Case Series 

**DOI:** 10.24908/pocus.v7iKidney.14997

**Published:** 2022-02-01

**Authors:** Ira Blau, Behdad Besharatian, Nathaniel Reisinger

**Affiliations:** 1 Penn Medicine Philadelphia, Pennsylvania

**Keywords:** Pelvic Kidney, Junctional Parenchymal Defect, Simple Renal Cyst, Incidentaloma, POCUS, Volunteer

## Abstract

A radiographic incidental finding (sometimes called an incidentaloma) is defined as a structure that is unintentionally found during an exam for an unrelated indication. The increased use of routine abdominal imaging is associated with a rising incidence in incidentalomas of the kidney 1. In one meta-analysis, 75% of renal incidentalomas were benign 2. However, the overall prevalence of incidental carcinomas is low at 0.2% 3. With the growing uptake of POCUS, healthy volunteers for clinical demonstrations may find themselves with new findings despite a lack of symptoms 4. Having an incidentaloma discovered during the course of a nephrology POCUS workshop is a unique experience. Herein we report our experiences of having incidentalomas discovered during the course of POCUS demonstrations.

## Case 1

**Figure 1:**
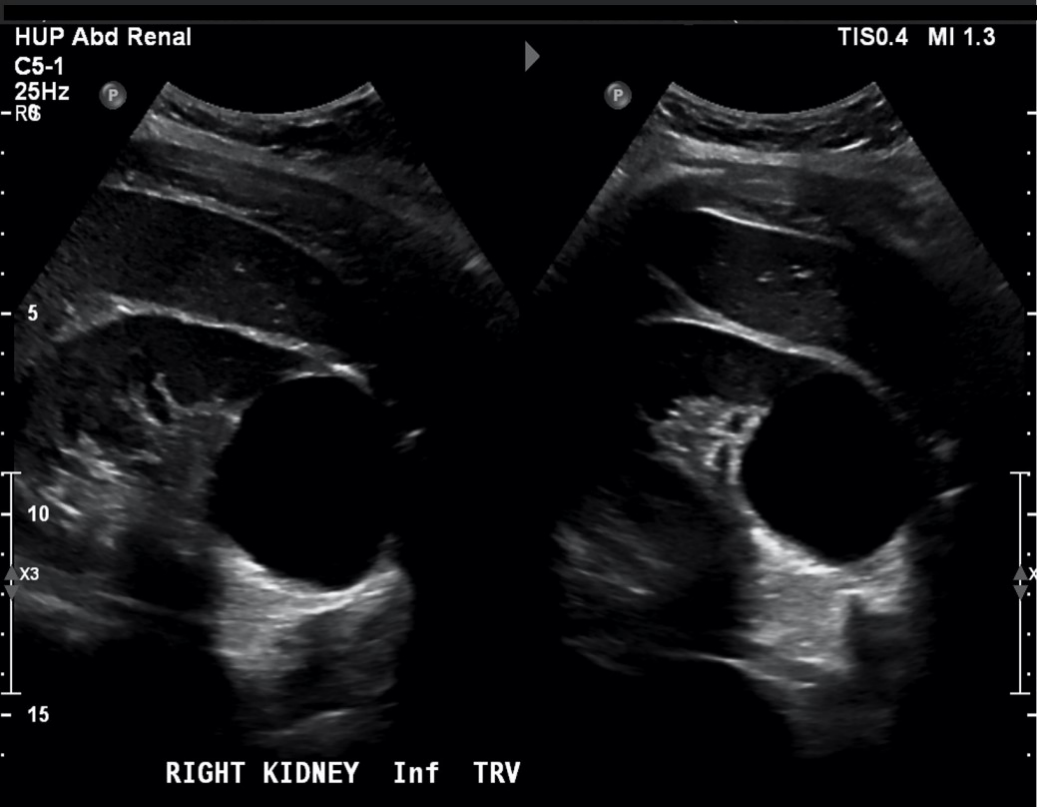
B mode sagittal (left) and transverse (right) imaging planes of right kidney from case 1. Note the thin-walled, anechoic 5 cm simple cyst at the inferior pole of the kidney with posterior acoustic enhancement.

**Figure 2:**
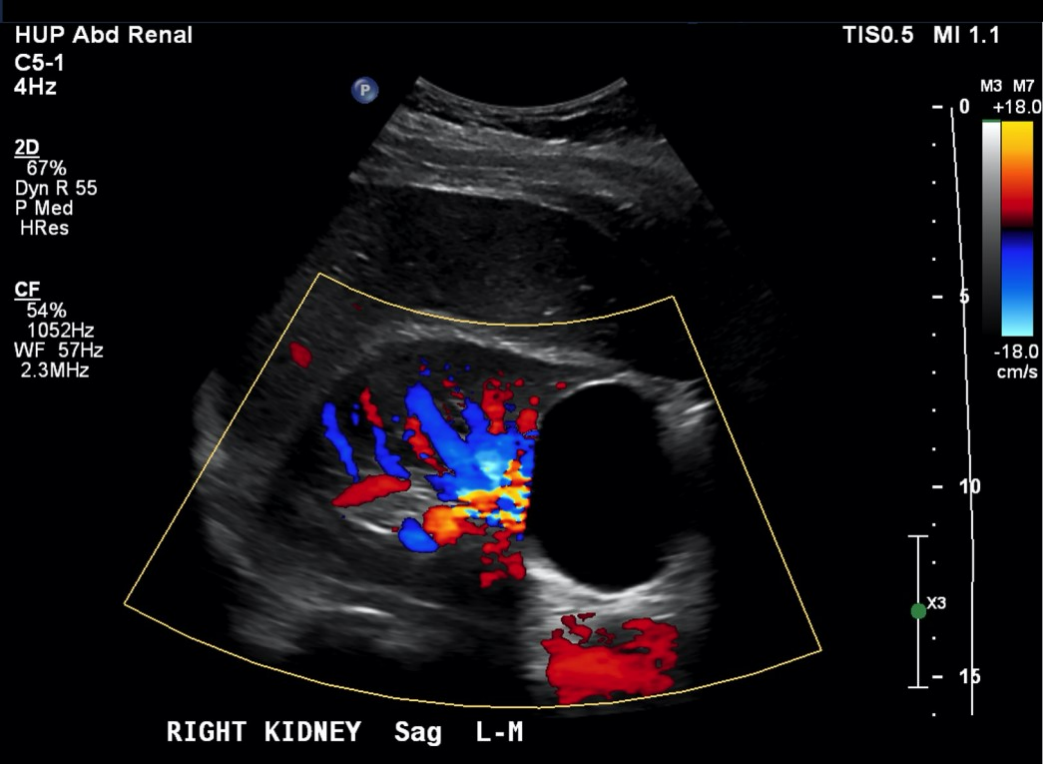
Doppler mode sagittal imaging plane of right kidney from case 1 demonstrating patent renal vasculature and absence of flow within the cyst.

A 31-year-old male nephrology fellow with history of intraocular hypertension controlled on latanoprost volunteered to serve as a model during a POCUS workshop as part of nephrology fellowship. He first learned the overall technique on an ultrasound simulator and proceeded to self-scan to obtain more practice. Standardized patients were not used due to group restrictions related to the SARS-CoV-2 pandemic. During the course of the examination, he identified a 5 cm anechoic structure with posterior acoustic enhancement at the inferior pole of his right kidney. The anechoic structure was well-circumscribed, thin-walled, and homogenous—lacking in internal echoes. The fellow asked for help from the attending nephrologist who also identified the structure as a simple cyst and ordered a referral study for follow up. A referral ultrasound study confirmed a 5 cm simple cyst at the inferior pole of the right kidney (Figure 1, 2). Serum creatinine was previously normal at 1.0 mg/dL. The fellow’s reaction was one of increased confidence in correctly identifying the structure, but a lurking feeling of unease that ethical issues should be considered in using volunteers for such programs.

Renal cysts are a very common finding on ultrasound occurring in more than 1 in 10 patients. Simple renal cysts appear as thin-walled anechoic structures with posterior acoustic enhancement. Simple cysts are benign, but increasing cyst complexity can be categorized according to the Bosniak Classification System with features such as septae, calcifications, vascularity, or solid components associated with increased risk of malignancy 5.

## Case 2 

**Figure 3:**
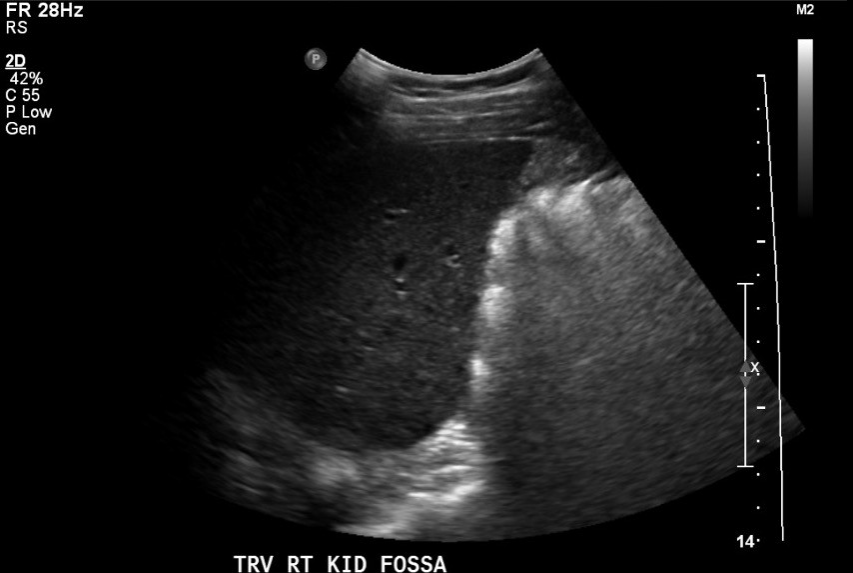
Sagittal imaging plane of right kidney fossa from case 2 demonstrating liver, but no kidney present.

**Figure 4:**
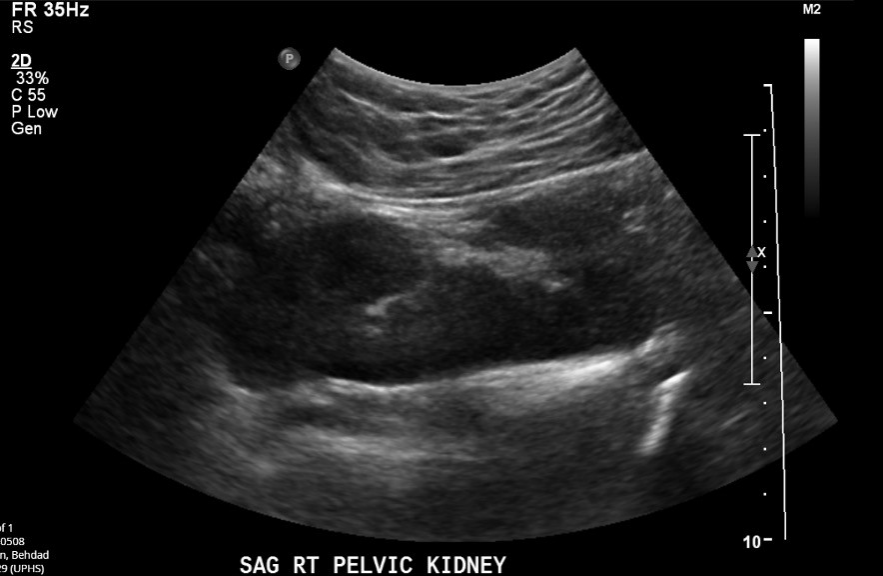
Oblique imaging plane of right iliac fossa from case 2 demonstrating the presence of renal parenchyma in this ectopic location.

**Figure 5:**
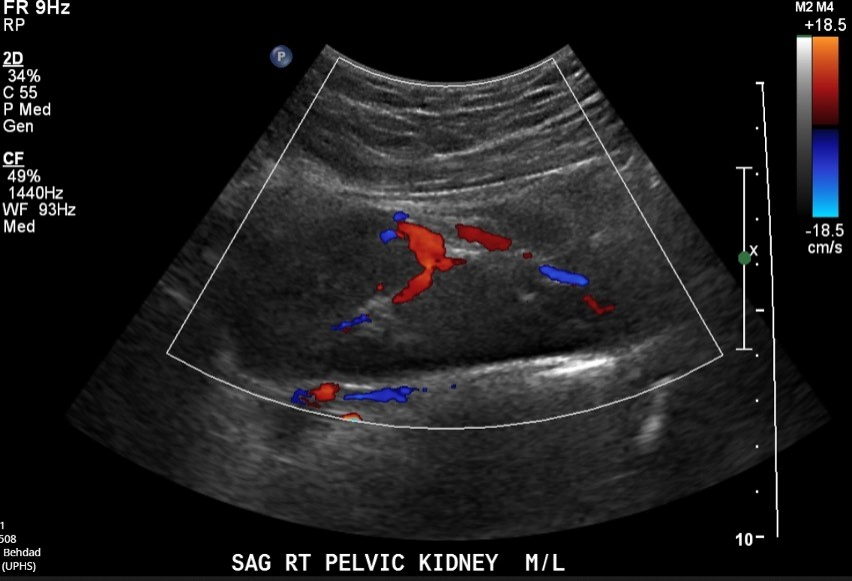
Doppler mode oblique imaging plane of right iliac fossa from case 2 demonstrating the presence of patent renal vasculature within the renal parenchyma in this ectopic location.

A healthy 32-year-old male nephrology fellow volunteered to serve as a model demonstrating renal imaging for a class of undergraduate medical students. The sonographer correctly identified the right renal fossa and liver but could not identify the right kidney. The sonographer quickly moved on to the left side and demonstrated a normal left kidney. The absence of right kidney was communicated to the experienced attending nephrologist and the class of medical students. The attending nephrologist sent the nephrology fellow for a referral study which again demonstrated an empty right renal fossa (Figure 3) and normal left kidney. Ultimately a pelvic kidney was identified in the right iliac fossa with patent vasculature and present right ureteral jet (Figure 4, 5). Serum creatinine was noted to be at the high end of normal at 1.3 mg/dL. The fellow later recalled that he had episodes of gross hematuria as a child following participation in sports. His overall reaction to the episode was bemusement and interest in the embryologic processes that led to this aberrantly placed kidney.

Pelvic kidneys occur in approximately 1 in 500 births and result from the failure of the ascent of the kidney from its embryological origin during the 9^th^ week. Pelvic kidneys are generally asymptomatic and are typically only discovered incidentally, but can be associated with other genitourinary malformations or complications such as obstructing nephrolithiasis. 6.

## Case 3

**Figure 6:**
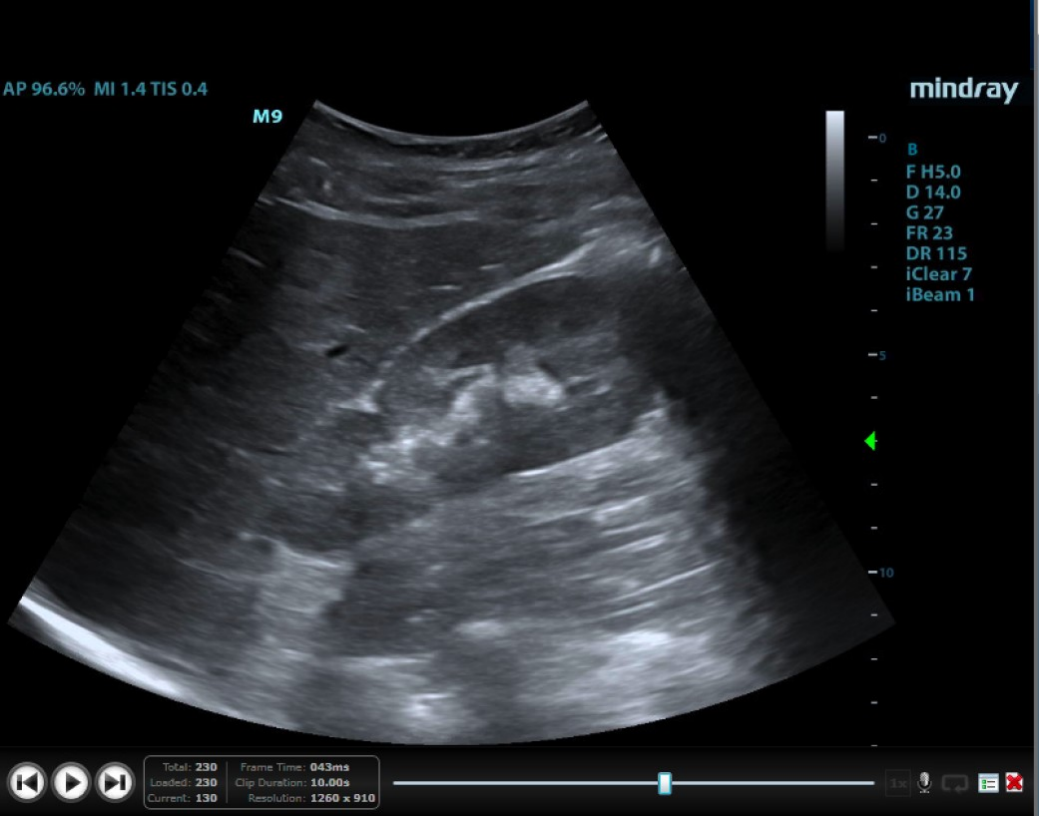
B mode sagittal imaging plane of right kidney demonstrating junctional parenchymal defect with characteristic “shark’s tooth” appearance. Note overlying liver isoechoic to kidney cortex.

A healthy 33-year-old male nephrology fellow scanned his own kidneys and bladder to generate normal images for a seminar on kidney and bladder ultrasound for nephrology fellows and attendings. He discovered a junctional parenchymal defect on his right kidney which he recognized by its distinctive appearance from a prior kidney ultrasound seminar (Figure 6). The kidneys and bladder were otherwise without abnormalities. A serum creatinine had been normal at 1.0 mg/dL on routine blood work one year prior. Referral imaging confirmed the finding of junctional parenchymal defect. He included the gathered images in the lecture as planned and later included the finding in a case series on incidental findings. His overall reaction was one of pleasure and self-satisfaction for correctly identifying the anatomical variant and at the opportunity to publish the finding in a peer-reviewed journal. 

A junctional parenchymal defect (JPD) is a triangular area hyperechoic to the renal parenchyma that can be mistaken for scar or mass. JPDs are most commonly seen in the upper pole of the right kidney representing an extension of the sinus fat and embryologically arising from fusion of embryologic renal precursors. Continuity of the JPD with renal sinus fat differentiates them from other pathologic lesions 7.

## Discussion

We reported three incidentalomas occurring in healthy nephrology fellows volunteering for POCUS workshops. In all three cases the findings were benign and represented the typical spectrum of findings on kidney ultrasound. This case series is limited in scope due to the small number of patients and demographic homogeneity. Loss of confidentiality in the case of an incidentally discovered finding remains a concern. Given these concerns we implemented a protocol faculty similar to that put forth by Siegel-Richman and Kendall 4. Namely, volunteers, whether students or standardized patients, have the right to have such incidental findings remain confidential, as well as the option to either pursue or refuse further diagnostic testing. Instructors and sonographers, for their part, should respect the wishes of the participants but remain vigilant and, regardless of their insurance status, recommend further testing and, if indicated, medical attention without delay. 

## Statement of ethics approval/consent

Authors state that they have no conflicts of interest. Authors attest that they give their consent for reproduction of the included images. 
